# Long-Acting Glucagon-Like Peptide-1 Receptor Agonists Suppress Voluntary Alcohol Intake in Male Wistar Rats

**DOI:** 10.3389/fnins.2020.599646

**Published:** 2020-12-23

**Authors:** Vincent N. Marty, Mehdi Farokhnia, Joseph J. Munier, Yatendra Mulpuri, Lorenzo Leggio, Igor Spigelman

**Affiliations:** ^1^Laboratory of Neuropharmacology, Section of Oral Biology, School of Dentistry, University of California, Los Angeles, Los Angeles, CA, United States; ^2^Clinical Psychoneuroendocrinology and Neuropsychopharmacology Section, Translational Addiction Medicine Branch, National Institute on Drug Abuse Intramural Research Program and National Institute on Alcohol Abuse and Alcoholism Division of Intramural Clinical and Biological Research, National Institutes of Health, Bethesda, MD, United States; ^3^Center on Compulsive Behaviors, National Institutes of Health, Bethesda, MD, United States; ^4^Johns Hopkins Bloomberg School of Public Health, Johns Hopkins University, Baltimore, MD, United States; ^5^Center for Alcohol and Addiction Studies, Department of Behavioral and Social Sciences, Brown University, Providence, RI, United States; ^6^Medication Development Program, National Institute on Drug Abuse Intramural Research Program, National Institutes of Health, Baltimore, MD, United States; ^7^Division of Addiction Medicine, Department of Medicine, School of Medicine, Johns Hopkins University, Baltimore, MD, United States; ^8^Department of Neuroscience, Georgetown University Medical Center, Washington, DC, United States

**Keywords:** glucagon-like peptide-1, liraglutide, semaglutide, alcohol intake, GPR119, dipeptidyl peptidase-4, rat

## Abstract

Alcohol use disorder (AUD) is a chronic relapsing condition characterized by compulsive alcohol-seeking behaviors, with serious detrimental health consequences. Despite high prevalence and societal burden, available approved medications to treat AUD are limited in number and efficacy, highlighting a critical need for more and novel pharmacotherapies. Glucagon-like peptide-1 (GLP-1) is a gut hormone and neuropeptide involved in the regulation of food intake and glucose metabolism via GLP-1 receptors (GLP-1Rs). GLP-1 analogs are approved for clinical use for diabetes and obesity. Recently, the GLP-1 system has been shown to play a role in the neurobiology of addictive behaviors, including alcohol seeking and consumption. Here we investigated the effects of different pharmacological manipulations of the GLP-1 system on escalated alcohol intake and preference in male Wistar rats exposed to intermittent access 2-bottle choice of 10% ethanol or water. Administration of AR231453 and APD668, two different agonists of G-protein receptor 119, whose activation increases GLP-1 release from intestinal L-cells, did not affect voluntary ethanol intake. By contrast, injections of either liraglutide or semaglutide, two long-acting GLP-1 analogs, potently decreased ethanol intake. These effects, however, were transient, lasting no longer than 48 h. Semaglutide, but not liraglutide, also reduced ethanol preference on the day of injection. As expected, both analogs induced a reduction in body weight. Co-administration of exendin 9-39, a GLP-1R antagonist, did not prevent liraglutide- or semaglutide-induced effects in this study. Injection of exendin 9-39 alone, or blockade of dipeptidyl peptidase-4, an enzyme responsible for GLP-1 degradation, via injection of sitagliptin, did not affect ethanol intake or preference. Our findings suggest that among medications targeting the GLP-1 system, GLP-1 analogs may represent novel and promising pharmacological tools for AUD treatment.

## Introduction

Alcohol use disorder (AUD) is a chronic relapsing condition characterized by compulsive alcohol-seeking, loss of control in limiting alcohol intake, and the emergence of a negative emotional state that leads to dependence on alcohol and serious detrimental health consequences (Rehm et al., 2009; Koob, 2015). The Food and Drug Administration (FDA) approved medications to treat AUD have shown efficacy, but their effect sizes are sub-optimal. Therefore, there is a critical need for investigating new pharmacotherapies targeting alternative pathways involved in the neurobiology of AUD (Klein, 2016; Farokhnia et al., 2019a; Witkiewitz et al., 2019).

Glucagon-like peptide-1 (GLP-1) is a gut neuropeptide hormone involved in the regulation of food intake and glucose homeostasis via GLP-1 receptors (GLP-1Rs). GLP-1 is produced both in the periphery, mainly by intestinal L-cells (Novak et al., 1987), and in several brain regions including the nucleus tractus solitarius (NTS), mainly by preproglucagon (PPG) neurons (Larsen et al., 1997; Merchenthaler et al., 1999). *In vivo*, the half-life of GLP-1 is very short (1.5 min following intravenous dosing and 1.5 h following subcutaneous dosing in humans), mainly due to degradation by the dipeptidyl peptidase-4 (DPP-4) enzyme (Deacon et al., 1995a, b). GLP-1R is a member of the secretin-like class B family of G-protein coupled receptors (GPCRs) expressed in various peripheral tissues, including vagal afferents, hepatic portal system and pancreatic β-cells, where they regulate food intake and glycemia by delaying gastric emptying, decreasing glucagon release and glucose-dependent stimulation of insulin secretion (Orskov et al., 1988; Flint et al., 2001; Kanoski et al., 2011). The critical role of GLP-1 in glucose homeostasis, food intake, and energy metabolism has led to the development of multiple FDA-approved GLP-1 analogs, such as exenatide, liraglutide, and semaglutide, for type 2 diabetes treatment (Agersø et al., 2002; Estall and Drucker, 2006; Eng et al., 2014; Lau et al., 2015). Liraglutide is also approved for the treatment of obesity. To overcome the rapid enzymatic degradation of GLP-1 and to prolong GLP-1R activation, liraglutide and semaglutide were designed by adding fatty-acid chains to the GLP-1 peptide, enhancing their binding affinity to albumin and protecting them against DPP-4 enzymatic degradation and renal filtration, while preserving their GLP-1R potency (Knudsen and Lau, 2019). Due to their prolonged half-lives, liraglutide and semaglutide are referred to as long-acting GLP-1 analogs, and are prescribed once daily and once weekly, respectively (Knudsen and Lau, 2019).

In the central nervous system (CNS), GLP-1Rs are expressed in several brain regions involved in regulating metabolism and energy balance, with preferential localization in inhibitory GABAergic neurons over excitatory glutamatergic neurons (Hayes et al., 2010; Cork et al., 2015; Fortin et al., 2020; Graham et al., 2020). In the hypothalamus, activation of GLP-1Rs has been shown to regulate food intake, glycemia, and stress responses (Kinzig et al., 2003; Sandoval et al., 2008; Hayes et al., 2010; Ghosal et al., 2017). GLP-1Rs are also found in reward-related brain regions that regulate appetitive and consummatory behaviors, such as the nucleus accumbens (NAc), ventral tegmental area (VTA), and amygdala (Göke et al., 1995; Alvarez et al., 1996; Merchenthaler et al., 1999; Cork et al., 2015; Graham et al., 2020). Recently, rodent studies have shown that GLP-1 analogs reduce the rewarding effects of several drugs of abuse, including alcohol, primarily via activation of central GLP-1Rs (Egecioglu et al., 2013a, b; Shirazi et al., 2013; Suchankova et al., 2015; Sirohi et al., 2016; Vallöf et al., 2016, 2019b). In humans, genetic variation in *GLP-1R* was found to be associated with increased risk of AUD, increased alcohol self-administration in a laboratory setting, and brain activity when being notified about the receipt of a monetary reward (Suchankova et al., 2015). Hence, GLP-1 analogs, already deemed to have a favorable safety profile in humans, are increasingly gaining attention as a novel therapeutic approach to treat AUD.

Recent studies have also renewed interest in the G protein-coupled receptor 119 (GPR119), a member of the class A of rhodopsin-like GPCRs, for its role in glucose homeostasis (Fredriksson et al., 2003; Soga et al., 2005). GPR119 is expressed in limited tissues, including the pancreatic islets and gastrointestinal tract. Activation of GPR119 via both direct actions on pancreatic β-cells and indirect actions on intestinal L-cells results in increased glucose-dependent insulin release and increased gene expression of proglucagon, a biosynthetic precursor of GLP-1, with subsequent release of endogenous GLP-1, glucose-dependent insulinotropic peptide, neurotensin, and peptide YY, leading to improvement of glucose tolerance in rodents and humans (Overton et al., 2006; Chu et al., 2007, 2008; Hansen et al., 2011; Katz et al., 2011; Lan et al., 2012; Chepurny et al., 2013; Mandøe et al., 2015; Hassing et al., 2016; Han et al., 2018; Matsumoto et al., 2018). Its restricted tissue distribution, relative selectivity in enhancing GLP-1 release, and tolerability of its agonists have made GPR119 an attractive therapeutic target for developing orally bioavailable agonists for type 2 diabetes treatment (Terauchi et al., 2018; Yang et al., 2018), and potentially for other related disorders.

In the present study we investigated the effects of different pharmacological manipulations of the GLP-1 system on escalated voluntary ethanol intake and preference, water intake, and body weight in male Wistar rats, in order to determine the potential utility of these compounds in the treatment of AUD. Specifically, we tested two GPR119 agonists, AR231453 and APD668, two long-acting GLP-1 analogs, liraglutide and semaglutide, a blocker of DPP-4, sitagliptin, and another peptide putatively working as a GLP-1R antagonist, exendin 9-39 (Ex9-39).

## Materials and Methods

### Animals

All experiments were performed in accordance with the guidance of the National Institutes of Health on animal care and use and the University of California, Los Angeles, Animal Research Committee. All rats were housed individually in the vivarium under a 12h light/dark cycle (lights on at 6AM) and had *ad libitum* access to food and water during the entire experiment. Two distinct cohorts of rats were used. Rats were included in the analyses only if the average ethanol preference from the last 3 presentations prior to vehicle administration reached 45% or higher. In the first cohort, 12 out of 17 rats reached this criterion. Thus, a total of 12 male Wistar rats (Envigo) weighing 260–300 g at the start of the experiment were used in the first cohort to study the effects of AR231453, APD668, liraglutide and semaglutide (administered alone and in combination with exendin 9-39), and sitagliptin. In the second cohort, 13 out of 15 rats reached the criterion mentioned above. However, one rat was identified as an outlier (water intake), using the ROUT method combining robust regression and outlier removal with *Q* = 1% (Motulsky and Brown, 2006), and therefore was removed from the study. Thus, a total of 12 male Wistar rats weighing 270–290 g at the start of the experiment were used in the second cohort to study the effects of exendin 9-39 alone.

### Intermittent Access 2-Bottle Choice Drinking Paradigm

Ethanol (EtOH, 95%, Decon Labs Inc., King of Prussia, PA, United States), meeting the United States Pharmacopeia (USP) specifications, was used to make all EtOH-containing solutions. The intermittent access to 2-bottle choice (IA2BC) drinking paradigm has been previously described (Simms et al., 2008; Meyer et al., 2013). This model leads rats to gradually escalate EtOH intake to high levels (4–6 g/kg/24h) without resorting to sucrose fadeout procedures or forced EtOH administration (Wise, 1975; Cippitelli et al., 2012). The IA2BC paradigm provides a platform to address multiple aspects of alcohol abuse in a rat model, including transition from social-like drinking to excessive alcohol consumption, binge drinking, alcohol seeking, relapse, and neuroadaptations related to excessive alcohol intake (Carnicella et al., 2014). Although some labs, including ours, report minimal alcohol deprivation effects (Simms et al., 2008; Meyer et al., 2013), others report marked withdrawal behaviors, such as anxiety-like symptoms and hyperalgesia after withdrawal from > 8 weeks of IA2BC (Kang et al., 2019; Li et al., 2019; Fu et al., 2020). Peak plasma EtOH concentrations obtained following 30 min of IA2BC 20% EtOH range between 4 and 93 mg/dL in Wistar rats (Simms et al., 2008); with a blood EtOH concentration of > 80 mg/dL meeting the criteria of the National Institute on Alcohol Abuse and Alcoholism (NIAAA) for binge drinking in humans. The model also has some predictive validity, e.g., the FDA-approved medication acamprosate attenuates drinking in the IA2BC but not in continuous access models (Simms et al., 2008; Cippitelli et al., 2012). All fluids were presented in 250-ml graduated plastic bottles with stainless steel low-leak drinking spouts accessible to rats through the top of their home cage. Rats were given access to 1 bottle of drinking water and 1 bottle of EtOH (10%, w/v) solution for a 24-h period on Mondays, Wednesdays, and Fridays. Bottles were weighed at the beginning of each 24-h drinking period, at approximately 1 h before the dark cycle (pre-injection). Measurements were taken to the nearest 1/100 g. The weight of each rat was also measured at the start of the 24-h drinking period and used to calculate the grams of solution consumed per kilogram of body weight per 24-h drinking session (g/kg/day). Preference for EtOH was calculated as the ratio of EtOH-containing solution over the total fluid consumed in a 24-h drinking session. Experimental EtOH-containing solutions were prepared in drinking water provided by UCLA veterinary staff. Upon completion of each drinking session, the EtOH-containing solution was replaced with a second water bottle until the next presentation. The rats had unlimited access to 2 bottles of water over the weekend after the 24-h measurements were taken on Saturday. Bottle placement was alternated each drinking session to control for side preferences. EtOH presentations were made until after the rats had reached a steady high level of drinking before drug administration. After reaching steady high level of drinking, rats were habituated to be immobilized in a plastic cone (DC 200, Braintree Scientific, Inc., Braintree, MA, United States) for about 5 s, just prior to EtOH presentation, in order to habituate them to the condition experienced during drug or vehicle injection. For injections, rats were immobilized in a plastic cone for the entire duration of the injection (∼5 s). Effects of drugs on body weight were determined by comparing the body weight measured during the day of injection (pre-injection) to the body weight measured at subsequent post-injection time-points.

### Study Drugs

All drugs were injected intraperitoneally between 4 and 5 PM, just before the start of the IA2BC EtOH presentation. Doses were chosen based on each drug’s EC_50_/IC_50_ or their reported effectiveness at modulation of EtOH intake. Vehicle was composed of DMSO:Tween 80:saline at 1:1:10 ratio. Two different GPR119 agonists, AR231453 (Millipore Sigma, Burlington, MA, United States) and APD668 (AOBIOUS, Gloucester, MA, United States), were used to stimulate GLP-1 release (Semple et al., 2008, 2011). AR231453 was dissolved in vehicle to a final dilution of 4 mg/ml and was injected at a concentration of 3 mg/kg. To overcome potential solubility issues, APD668 was dissolved in two different vehicles (DMSO or polyethylene glycol 400 – PEG-8) to a final dilution of 8 mg/ml and was injected at a concentration of 6 mg/kg. Two long-acting GLP-1 analogs, liraglutide (Cayman Chemical, Ann Arbor, MI, United States) and semaglutide (MedChem Express, Monmouth Junction, NJ, United States) were used (Larsen et al., 2001; Lau et al., 2015). Liraglutide and semaglutide were dissolved in vehicle to a final dilution of 1 mg/ml and injected at a concentration of 0.1 mg/kg. Exendin 9-39 (Ex9-39; Bachem, Torrance, CA, United States), a GLP-1R antagonist (Schepp et al., 1994), was dissolved in saline to a final dilution of 0.13 mg/ml and was injected at a concentration of 0.1 mg/kg. When co-administered with liraglutide (0.1 mg/kg) or semaglutide (0.1 mg/kg), Ex9-39 was injected at a concentration of 0.15 mg/kg or 0.19 mg/kg, respectively. Sitagliptin phosphate (Millipore Sigma, Burlington, MA, United States), an inhibitor of DPP-4, was dissolved in saline to a final dilution of 33.3 mg/ml and was injected at a concentration of 25 mg/kg.

### Statistical Analysis

Statistical analyses were performed using Prism 7 (GraphPad Software Inc., La Jolla, CA, United States) and data were expressed as mean ± standard error of the mean (SEM). All data sets were tested for outliers using the ROUT method (Motulsky and Brown, 2006). Two-way repeated-measures analyses of variance (RM ANOVA) was used to assess significance of the effects of treatment (vehicle vs. drug), time-point (baseline, injection day, + 2 days) and treatment × time-point interaction on EtOH intake, EtOH preference, and water intake. Given that the treatment × time-point interaction effect was our primary outcome of interest, when the interaction term was found to be significant, Tukey’s *post hoc* test was used to further identify statistical significance between treatments and/or time-points. For the two-way RM ANOVAs, the baseline time-point represents the average of 3 presentations prior to vehicle/drug injection. The injection day time-point represents the value of EtOH intake, EtOH preference or water intake measured 24-h post-injection. The detailed two-way RM ANOVA tables, including treatment, time-point, and treatment × time-point interaction effects are presented in Supplementary Tables 1–3. Statistical power was calculated each time a two-way RM ANOVA revealed a significant treatment × time-point interaction effect, using G^∗^Power (version 3.1.9.6, Ute Clames), and was reported only when the value was below 0.8. We found two instances where the statistical power was lower than 0.8 (EtOH intake under APD668 (DMSO) and Ex9-39), and report these results as potentially false positives. Because body weight was consistently increasing as the cohort’s aged, we used one-way RM ANOVAs, followed by Dunnett’s *post hoc* tests, to assess the effect of each drug on body weight. For these one-way RM ANOVAs, the pre-injection time-point represents the body weight measured 1 h prior to vehicle/drug injection on the day of injection, which was compared to the body weight measured at subsequent post-injection time-points. For AR231453 and APD668 experiments, the pre-injection body weight was compared to two post-injection time-points (+ 2-day and + 5-day). For liraglutide and semaglutide experiments (with and without Ex9-39), the pre-injection body weight was compared to five post-injection time-points (+ 2-day, + 5-day, + 7-day, + 9-day, and + 12-day) to assess the time course of recovery from the drug-induced weight loss. For sitagliptin experiments, the pre-injection body weight was compared to two post-injection time-points (+ 2-day and + 7-day; body weights measured from the + 5-day time-point were accidently lost). For the Ex9-39 alone experiment, the pre-injection body weight was compared to two post-injection time-points (+ 2-day and + 5-day). A value of *p* < 0.05 was considered statistically significant (^∗^*p* < 0.05, ^∗∗^*p* < 0.01, ^∗∗∗^*p* < 0.001).

## Results

### Activation of GPR119 Does Not Affect Ethanol Intake

**FIGURE 1:**
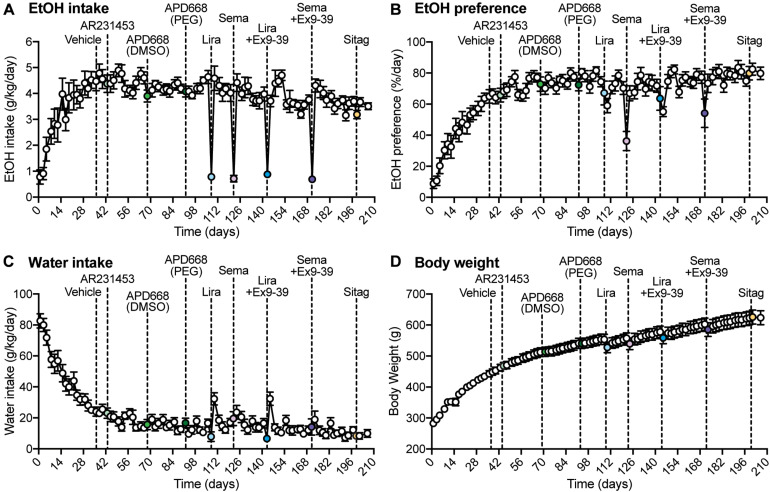
Overall measurements of EtOH intake, EtOH preference, water intake, and body weight during the experiment. **(A–D)** Dashed lines represent the time of injection of each drug. Vehicle was injected at day 36. AR2314553 (light green circle) was injected at day 43. APD668 (DMSO) (green circle) was injected at day 68. APD668 (PEG) (dark green circle) was injected at day 92. Liraglutide (Lira, light blue circle) was injected at day 108. Semaglutide (Sema, lavender circle) was injected at day 122. Liraglutide + Ex9-39 (Lira + Ex9-39, blue circle) was injected at day 143. Semaglutide + Ex9-39 (Sema + Ex9-39, purple circle) was injected at day 171. Sitagliptin (Sitag, yellow circle) was injected at day 199. **(A)** Time course of the average EtOH intake (g/kg/day) (*n* = 12 rats). EtOH intake was measured on Mondays, Wednesdays, and Fridays from day 1 to 206. From day 1 to 29, rats progressively drank higher amounts of EtOH 10% (v/w) until reaching a plateau. **(B)** Time course of the average EtOH preference (%/day) (*n* = 12 rats). EtOH preference was calculated as the ratio of EtOH-containing solution over the total fluid consumed in a 24-h drinking session (see section “Materials and Methods”). EtOH preference progressively increased until reaching a plateau. **(C)** Time course of the average water intake (g/kg/day) (*n* = 12 rats). Water intake progressively decreased until reaching a plateau. **(D)** Time course of the average body weight (g) (*n* = 12 rats). The average body weight was 280 ± 2.96 g at day 1, and progressively increased throughout the experiment to reach an average of 623 ± 22.56 g at day 206.

**FIGURE 2:**
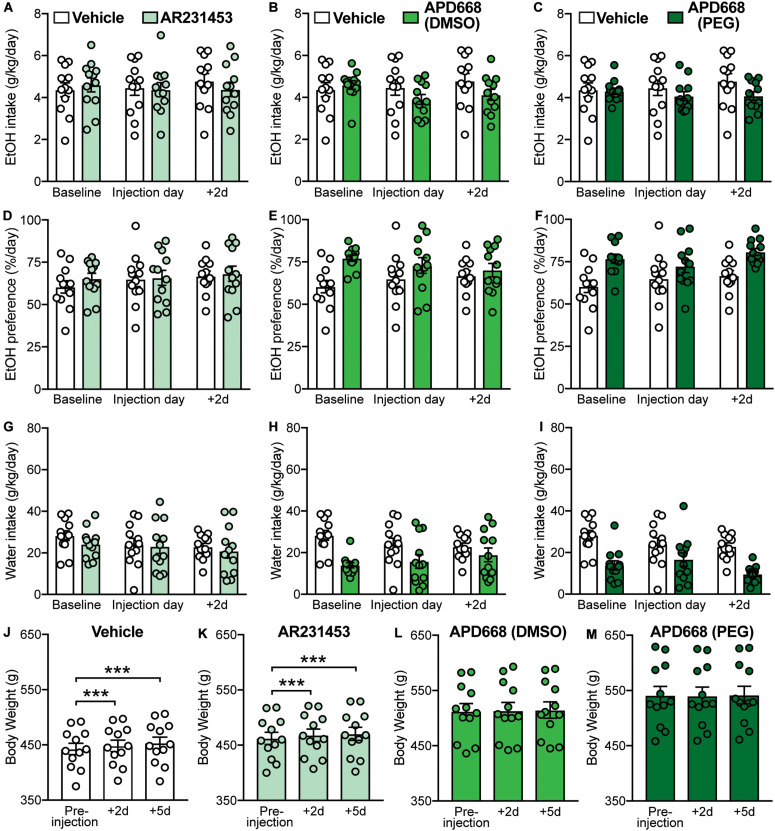
Effects of GPR119 agonists [AR231453, APD668 (DMSO), and APD668 (PEG)], compared to vehicle, on EtOH intake, preference, water intake, and body weight. Bars and circles represent the mean and individual data points, respectively. **(A–I)** Baseline represents the average of 3 presentations prior to vehicle/drug injection. **(A–C)** No significant treatment × time-point interaction effects on EtOH intake were found (note: the interaction term for APD668 (DMSO) was statistically significant, but due to low power, these results were concluded to be potentially false positive). **(D–F)** No significant treatment × time-point interaction effects on EtOH preference were found. **(G–I)** No significant treatment × time-point interaction effects on water intake were found. **(J,K)** Significant increases in body weight were observed under vehicle and AR231453, as shown by significant increases in body weight measured at 2-day (+ 2d) and 5-day (+ 5d) post-injection compared to the body weight measured on the day of injection (pre-injection). **(L,M)** No significant changes in body weight were observed under APD668 (DMSO) or APD668 (PEG), as shown by the lack of significant increases in body weight measured at 2-day (+ 2d) and 5-day (+ 5d) post-injection compared to the body weight measured on the day of injection (pre-injection). ***p < 0.001.

Activation of GPR119 can increase the release of plasma GLP-1 in rodents and humans (Chu et al., 2008; Hansen et al., 2011; Katz et al., 2011; Lan et al., 2012; Mandøe et al., 2015; Han et al., 2018; Matsumoto et al., 2018). Since GLP-1R activation has been shown to modulate EtOH consumption (Shirazi et al., 2013; Vallöf et al., 2016), we first investigated whether activation of GPR119 alters EtOH intake and preference, as well as water intake and body weight, using two different GPR119 agonists, AR231453 (3 mg/kg) or APD668 (6 mg/kg) (Figures 1A–D). For AR231453, the two-way RM ANOVAs did not find significant treatment × time-point interaction effects on EtOH intake (*F*(2,22) = 1.89, *p* = 0.17) (Figure 2A), EtOH preference (*F*(2,22) = 0.14, *p* = 0.87) (Figure 2D), or water intake (*F*(2,22) = 0.18, *p* = 0.83) (Figure 2G). For APD668 dissolved in DMSO, a significant treatment × time-point interaction effect was observed on EtOH intake (*F*(2,22) = 5.75, *p* = 0.0098) (Figure 2B), but due to low statistical power (power = 0.51), these results were concluded to be potentially false positive. Moreover, APD668 (DMSO) did not affect EtOH preference (treatment × time-point interaction: *F*(2,22) = 2.49, *p* = 0.11) (Figure 2E), or water intake (treatment × time-point interaction: *F*(2,22) = 2.85, *p* = 0.08) (Figure 2H). Consistent with these findings, APD668 dissolved in PEG did not affect EtOH intake (treatment × time-point interaction: *F*(2,22) = 2.79, *p* = 0.083) (Figure 2C), EtOH preference (treatment × time-point interaction: *F*(2,22) = 1.77, *p* = 0.19) (Figure 2F), or water intake (treatment × time-point interaction: *F*(2,22) = 2.44, *p* = 0.11 (Figure 2I).

As expected, and consistent with the overall trend as the rats aged (Figure 1D), body weight gain was observed following vehicle injection at 2-day (+ 2d) and 5-day (+ 5d) post-injection (*F*(2,22) = 67.27, *p* < 0.001; *post hoc* test, pre-injection vs. + 2d, *p* < 0.001; pre-injection vs. + 5d, *p* < 0.001) (Figure 2J). Similarly, increases in body weight was found at + 2d and + 5d following AR231453 injection (*F*(2,22) = 29.17, *p* < 0.001; *post hoc* test, pre-injection vs. + 2d, *p* < 0.001; pre-injection vs. + 5d, *p* < 0.001) (Figure 2K). In contrast, rats injected with APD668 (dissolved in either DMSO or PEG) did not gain weight following injections (APD668 (DMSO): *F*(2,22) = 0.7, *p* = 0.45; APD668 (PEG): *F*(2,22) = 1.1, *p* = 0.33) suggesting a putative effect of APD668 in body weight regulation (i.e., prevention of weight gain in this case) (Figures 2L,M).

### Activation of GLP-1 Receptor Reduces Ethanol Intake

**FIGURE 3:**
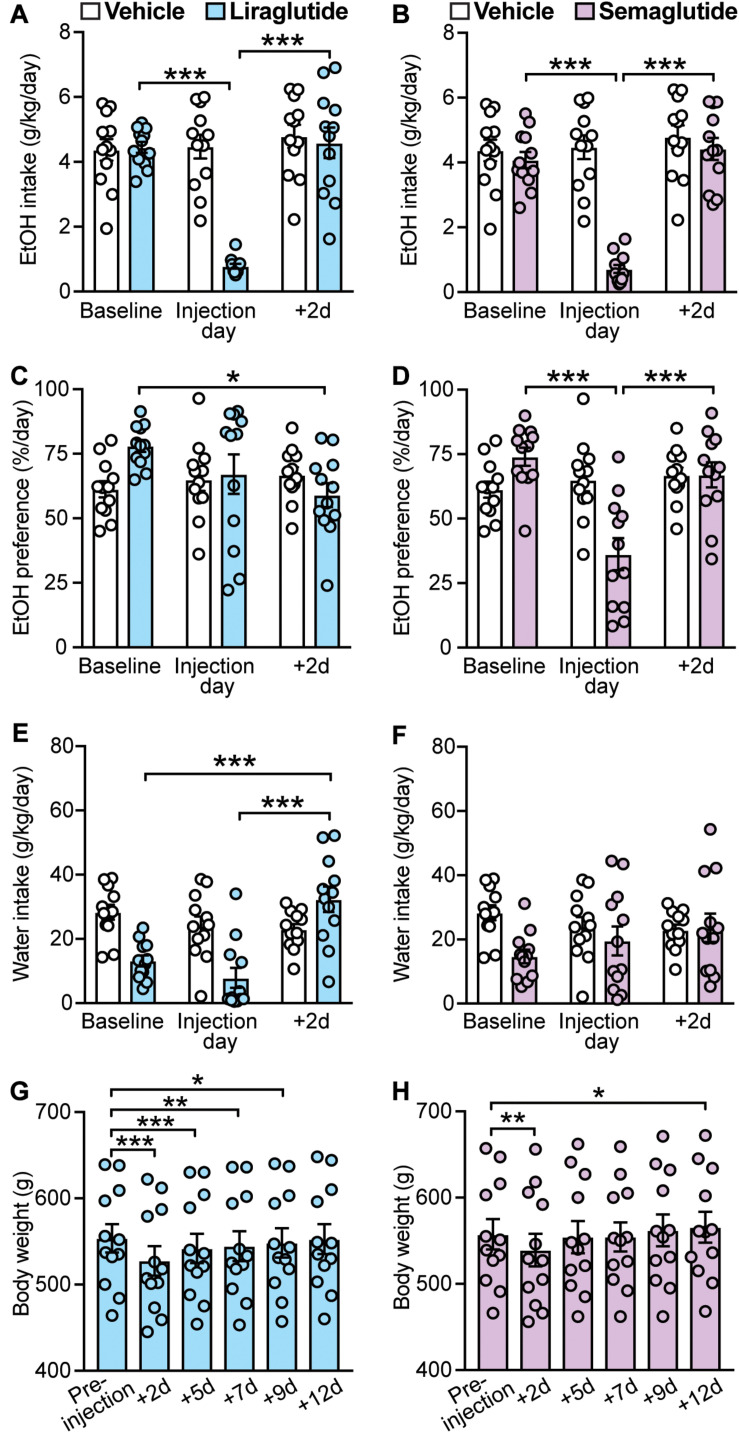
Effects of GLP-1 analogs (liraglutide and semaglutide), compared to vehicle, on EtOH intake, preference, water intake, and body weight. Bars and circles represent the mean and individual data points, respectively. **(A–F)** Baseline represents the average of 3 presentations prior to vehicle/drug injection. **(A)** A significant treatment × time-point interaction effect on EtOH intake was found for liraglutide; under liraglutide, EtOH intake at the injection day was lower than baseline and 2-day post-injection (+ 2d). **(B)** A significant treatment × time-point interaction effect on EtOH intake was found for semaglutide; under semaglutide, EtOH intake at the injection day was lower than baseline and + 2d. **(C)** A significant treatment × time-point interaction effect on EtOH preference was found for liraglutide; under liraglutide, EtOH preference at + 2d was lower than baseline. **(D)** A significant treatment × time-point interaction effect on EtOH preference was found for semaglutide; under semaglutide, EtOH preference at the injection day was lower than baseline and + 2d. **(E)** A significant treatment × time-point interaction effect on water intake was found for liraglutide; under liraglutide, water intake at + 2d was higher than baseline and the injection day. **(F)** No significant treatment × time-point interaction effect on water intake was found for semaglutide. **(G)** Significant changes in body weight were observed under liraglutide; body weight at + 2d, + 5d, + 7d, and + 9d was lower than the body weight measured prior injection on injection day (pre-injection). **(H)** Significant changes in body weight were observed under semaglutide; body weight at + 2d was lower, and at + 12d was higher, than the body weight measured prior injection on injection day (pre-injection). *p < 0.05, **p < 0.01, ***p < 0.001.

GLP-1R agonists have been shown to decrease voluntary EtOH intake in rodents suggesting a role for these ligands in the treatment of AUD (Shirazi et al., 2013; Vallöf et al., 2016). We found that both liraglutide (0.1 mg/kg) and semaglutide (0.1 mg/kg), two long-acting GLP-1 analogs, produced significant decreases in EtOH intake (liraglutide: treatment × time-point interaction: *F*(2,22) = 47.5, *p* < 0.001; *post hoc* test, baseline vs. liraglutide injection day: *p* < 0.001; semaglutide: treatment × time-point interaction: *F*(2,22) = 65.75, *p* < 0.001; *post hoc* test, baseline vs. semaglutide injection day: *p* < 0.001). Of note, the effects of liraglutide and semaglutide were transient, lasting no longer than the 2-day post-injection time-point (liraglutide: baseline vs. + 2d: *p* = 0.89; semaglutide: baseline vs. + 2d: *p* = 0.35) (Figures 3A,B).

Statistical analyses also showed a significant treatment × time-point interaction for the effects of liraglutide on EtOH preference (*F*(2,22) = 4.29, *p* = 0.03). However, *post hoc* testing only found a significance difference between baseline and 2-day post-injection time-points (baseline vs. liraglutide injection day: *p* = 0.18; baseline vs. + 2d: *p* = 0.011; liraglutide injection day vs. + 2d: *p* = 0.38) (Figure 3C). We also found that liraglutide significantly altered water intake with a significant increase at the 2-day post-injection time-point (treatment × time-point interaction: *F*(2,22) = 17.78, *p* < 0.001; *post hoc* test, baseline vs. + 2d: *p* < 0.001; liraglutide injection day vs. + 2d: *p* < 0.001) (Figure 3E). These data suggest that liraglutide-induced increase in water intake at + 2d, while not altering EtOH intake at this time-point (i.e., EtOH intake returned back to baseline), may be responsible for the decrease in EtOH preference observed at 2-day post-injection, rather than a direct effect on alcohol preference. By contrast, semaglutide significantly decreased EtOH preference at the injection day time-point (treatment × time-point interaction: *F*(2,22) = 14.01, *p* < 0.001; *post hoc* test, baseline vs. semaglutide injection day, *p* < 0.001; semaglutide injection day vs. + 2d, *p* < 0.001), without affecting water intake (treatment × time-point interaction: *F*(2,22) = 2.03, *p* = 0.16) (Figures 3D,F). These data suggest that while both GLP-1 analogs can reduce EtOH intake, semaglutide appears to be more selective for EtOH as it also reduced EtOH preference without affecting water intake.

Body weight was influenced by both GLP-1 analogs. Liraglutide induced long-lasting decreases in body weight up to 9 days post-injection (+ 9d) (*F*(5,55) = 101.2, *p* < 0.001; *post hoc* test, pre-injection vs. + 2d, *p* < 0.001; pre-injection vs. + 5d, *p* < 0.001; pre-injection vs. + 7d, *p* = 0.0012; pre-injection vs. + 9d, *p* = 0.014) (Figure 3G). By contrast, semaglutide induced only a transient decrease in body weight lasting no longer than 48 h, with a significant increase observed at 12 days post-injection (*F*(5,55) = 19.4, *p* < 0.001; *post hoc* test, pre-injection vs. + 2d, *p* = 0.005; pre-injection vs. + 12d, *p* = 0.02) (Figure 3H).

### GLP-1 Receptor Antagonism Does Not Prevent the Suppressing Effects of Liraglutide or Semaglutide on Ethanol Intake

**FIGURE 4:**
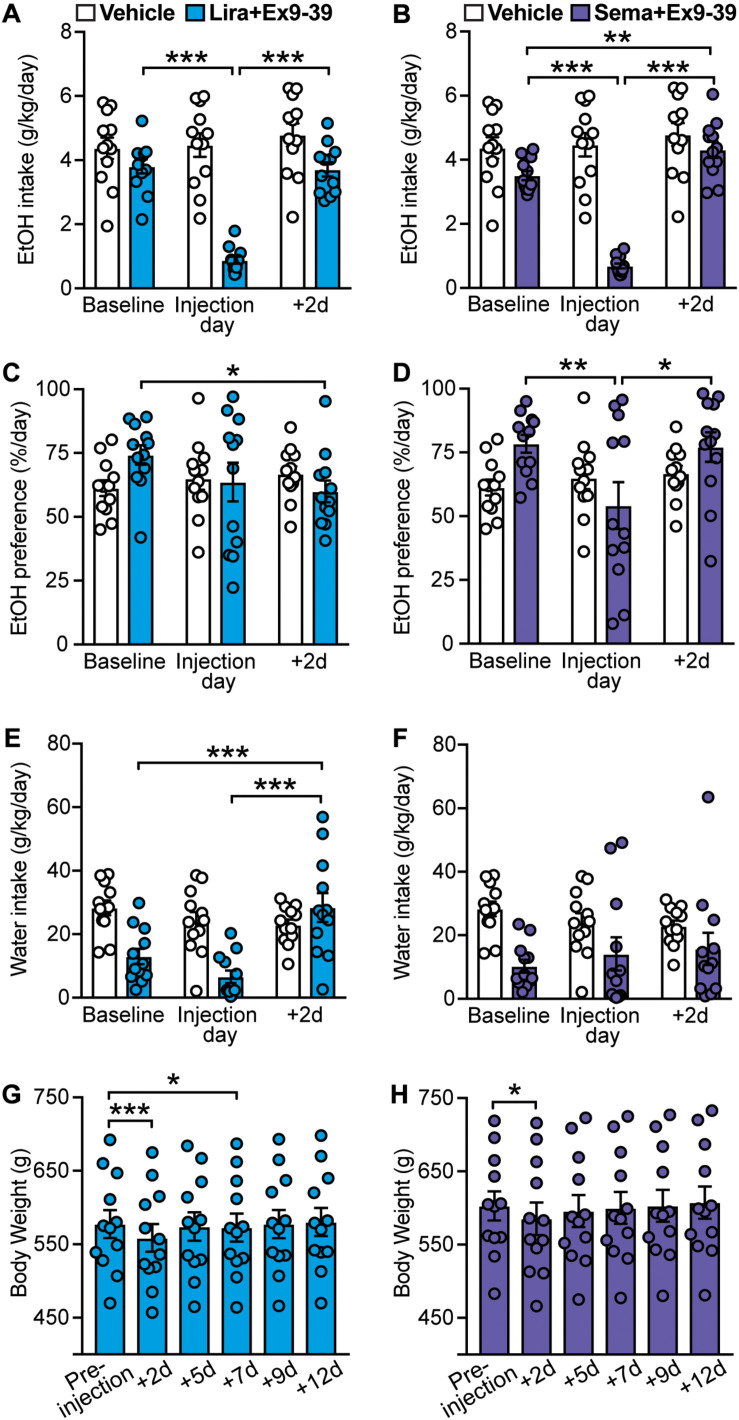
Effects of GLP-1 analogs (liraglutide and semaglutide) co-administered with a GLP-1R antagonist (Ex9-39), compared to vehicle, on EtOH intake, preference, water intake, and body weight. Bars and circles represent the mean and individual data points, respectively. **(A–F)** Baseline represents the average of 3 presentations prior to vehicle/drug injection. **(A)** A significant treatment × time-point interaction effect on EtOH intake was found for liraglutide + Ex9-39; under liraglutide + Ex9-39, EtOH intake at the injection day was lower than baseline and 2-day post-injection (+ 2d). **(B)** A significant treatment × time-point interaction effect on EtOH intake was found for semaglutide + Ex9-39; under semaglutide + Ex9-39, EtOH intake at the injection day was lower than baseline and + 2d, and EtOH intake at + 2d was higher than baseline. **(C)** A significant treatment × time-point interaction effect on EtOH preference was found for liraglutide + Ex9-39; under liraglutide + Ex9-39, EtOH preference at + 2d was lower than baseline. **(D)** A significant treatment × time-point interaction effect on EtOH preference was found for semaglutide + Ex9-39; under semaglutide + Ex9-39, EtOH preference at the injection day was lower than baseline and + 2d. **(E)** A significant treatment × time-point interaction effect on water intake was found for liraglutide + Ex9-39; under liraglutide + Ex9-39, water intake at + 2d was higher than baseline and the injection day. **(F)** No significant treatment × time-point interaction effect on water intake was found for semaglutide + Ex9-39. **(G)** Significant changes in body weight were observed under liraglutide + Ex9-39; body weight at + 2d, and + 7d was lower than the body weight measured on injection day (pre-injection). **(H)** Significant changes in body weight were observed under semaglutide + Ex9-39; body weight at + 2d was lower than the body weight measured on injection day (pre-injection). *p < 0.05, **p < 0.01, ***p < 0.001.

Ex9-39 has been shown to antagonize the effects of GLP-1R activation in rodents and humans (Thorens et al., 1993; Wang et al., 1995; Edwards et al., 1999; Vallöf et al., 2019a). Blockade of GLP-1R by co-administration of Ex9-39 did not prevent the decrease in EtOH intake induced by liraglutide (treatment × time-point interaction: *F*(2,22) = 49.22, *p* < 0.001; *post hoc* test, baseline vs. liraglutide + Ex9-39 injection day, *p* < 0.001; liraglutide + Ex9-39 injection day vs. + 2d, *p* < 0.001) (Figure 4A). Similarly, co-administration of Ex9-39 did not prevent the decrease in EtOH intake induced by semaglutide (treatment × time-point interaction: *F*(2,22) = 62.89, *p* < 0.001; *post hoc* test, baseline vs. semaglutide + Ex9-39 injection day, *p* < 0.001; baseline vs. + 2d, *p* = 0.005; semaglutide + Ex9-39 injection day vs. + 2d, *p* < 0.001) (Figure 4B).

Similar to liraglutide alone, liraglutide + Ex9-39 significantly decreased EtOH preference only at 2-day post-injection (treatment × time-point interaction: *F*(2,22) = 3.75, *p* = 0.04; *post hoc* test, baseline vs. + 2d, *p* = 0.03) (Figure 4C), which was concomitant with significant increase in water intake (treatment × time-point interaction: *F*(2,22) = 12.04, *p* < 0.001; *post hoc* test, baseline vs. + 2d, *p* < 0.001, liraglutide + Ex9-39 vs. + 2d, *p* < 0.001) (Figure 4E). Co-administration of Ex9-39 did not prevent the decrease in EtOH preference induced by semaglutide (treatment × time-point interaction: *F*(2,22) = 3.83, *p* = 0.037; *post hoc* test, baseline vs. semaglutide + Ex9-39, *p* = 0.009; semaglutide + Ex9-39 vs. + 2d, *p* = 0.015) (Figure 4D). Semglutide + Ex9-39 did not affect water intake (treatment × time-point interaction: *F*(2,22) = 1.1, *p* = 0.35) (Figure 4F).

Liraglutide + Ex9-39 induced a transient decrease in body weight (*F*(5,55) = 30.65, *p* < 0.001; *post hoc* test, pre-injection vs. + 2d, *p* < 0.001; pre-injection vs. + 7d, *p* = 0.02) (Figure 4G). Similarly, semaglutide + Ex9-39 induced a reduction in body weight at 2-day post-injection (*F*(5,55) = 18.5, *p* < 0.001; *post hoc* test, pre-injection vs. + 2d, *p* = 0.01) (Figure 4H).

### Inhibition of DPP-4 Does Not Affect Ethanol Intake

**FIGURE 5:**
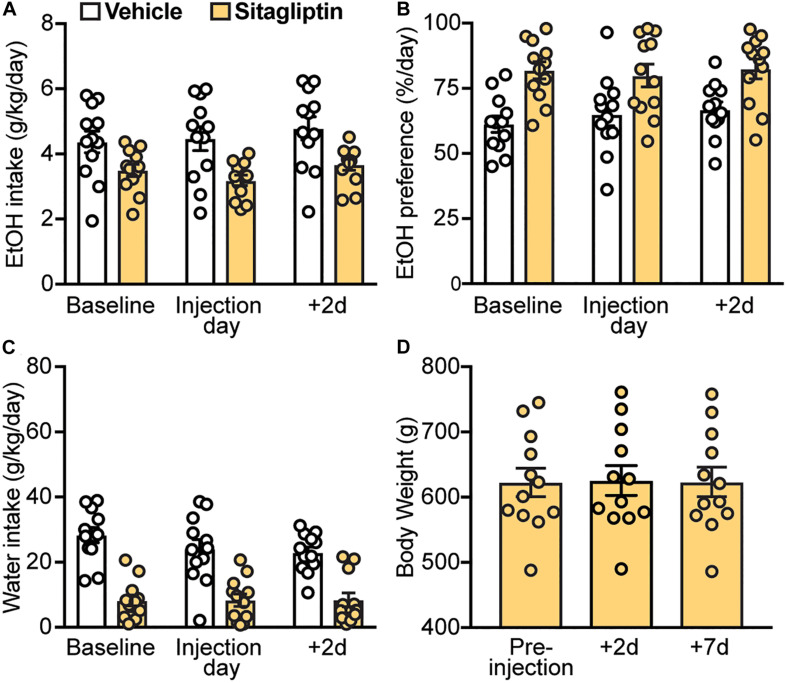
Effects of a DPP4 inhibitor (sitagliptin), compared to vehicle, on EtOH intake, preference, water intake, and body weight. Bars and circles represent the mean and individual data points, respectively. **(A–C)** Baseline represents the average of 3 presentations prior to vehicle/drug injection. No significant treatment × time-point interaction effects on EtOH intake, EtOH preference, or water intake were found. **(D)** No significant changes in body weight were observed.

Next, we investigated whether increasing endogenous GLP-1 levels by preventing its enzymatic degradation via DPP-4 could decrease EtOH intake and preference. Here, we found that sitagliptin did not affect EtOH intake (treatment × time-point interaction: *F*(2,22) = 0.95, *p* = 0.40) (Figure 5A), EtOH preference (treatment × time-point interaction: *F*(2,22) = 0.55, *p* = 0.58) (Figure 5B), or water intake (treatment × time-point interaction: *F*(2,22) = 1.32, *p* = 0.29) (Figure 5C). In addition, no changes in body weight were observed with sitagliptin (*F*(2,22) = 1.44, *p* = 0.26) (Figure 5D).

### GLP-1 Receptor Antagonism Does Not Affect Ethanol Intake

**FIGURE 6:**
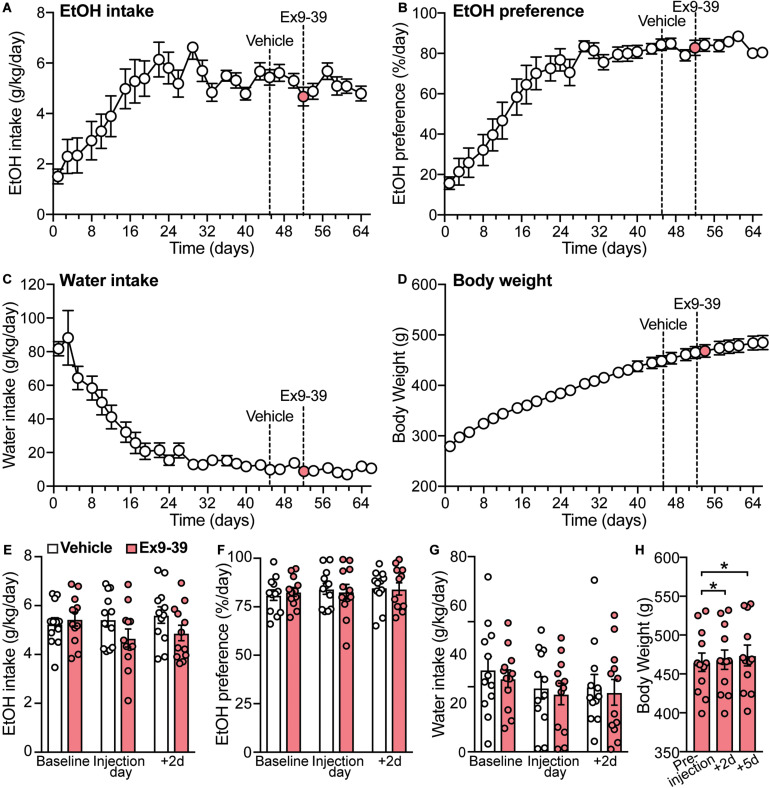
Overall measurements of EtOH intake, EtOH preference, water intake, and body weight for the second cohort of rats (*n* = 12). **(A–D)** Dashed lines represent the time of injection of vehicle (day 45) and Ex9-39 (day 52). **(E–G)** Effects of Ex9-39, compared to vehicle, on EtOH intake, preference, water intake; bars and circles represent the mean and individual data points, respectively. Baseline represents the average of 3 presentations prior to vehicle/drug injection. No significant treatment × time-point interaction effects on EtOH intake, EtOH preference, or water intake were found (note: the effect of the interaction term on EtOH intake was statistically significant, but due to low power, these results were concluded to be potentially false positive). **(H)** Effects of Ex9-39 on body weight. Significant changes in body weight were observed; body weight at + 2d and + 5d was higher than the body weight measured on injection day (pre-injection). *p < 0.05.

A previous study showed that a single dose of the GLP-1R antagonist Ex9-39 increased voluntary EtOH intake in rats (Shirazi et al., 2013). Considering these data and our findings that co-administration of Ex9-39 did not block the effects of liraglutide or semaglutide on EtOH intake, we investigated, in a different cohort of rats, whether injection of Ex9-39 alone, compared to vehicle, would affect EtOH intake, preference, water intake, and/or body weight (Figures 6A–D). A significant treatment × time-point interaction effect of Ex9-39 on EtOH intake was observed (*F*(2,22) = 3.8, *p* = 0.038) (Figure 6E), but due to low statistical power (power = 0.57), there results were concluded to be potentially false positive. Moreover, Ex9-39 did not affect EtOH preference (treatment × time-point interaction: *F*(2,22) = 0.19, *p* = 0.82) (Figure 6F), or water intake (treatment × time-point interaction: *F*(2,22) = 0.01, *p* = 0.99) (Figure 6G). We also found that body weight significantly increased at 2- and 5-day post-injection of Ex9-39 (*F*(2,22) = 8.56, *p* = 0.01; *post hoc* test, pre-injection vs. + 2d, *p* = 0.04; pre-injection vs. + 5d, *p* = 0.02) (Figure 6H).

## Discussion

Increasing evidence points to an important role of gut-brain peptides, including GLP-1, in modulating the biobehavioral correlates of alcohol use (Thorsell and Mathé, 2017; Jerlhag, 2018; Farokhnia et al., 2019b; von Holstein-Rathlou and Gillum, 2019; Hopf, 2020). Here, we found that GLP-1 analogs, liraglutide and semaglutide, potently, albeit transiently, decreased voluntary EtOH intake. Notably, semaglutide also reduced alcohol preference. Administration of Ex9-39, an inhibitor of GLP-1R, failed to prevent liraglutide- and semaglutide-induced decreases in EtOH intake. Sitagliptin, a blocker of DPP-4, the enzyme responsible for GLP-1 degradation, did not affect EtOH intake or preference. Similarly, activation of GPR119, which has been shown to promote endogenous GLP-1 release, had no effect on EtOH intake. Altogether, our findings support the hypothesis that direct pharmacological agonism of the GLP-1 receptor may represent an effective approach for AUD treatment.

Several recent studies in rodents have highlighted the role of the GLP-1 system in regulating addictive behaviors related to different drugs of abuse, including nicotine, cocaine, opioids, and alcohol, suggesting a new and promising pharmacotherapeutic target for drug addiction (Egecioglu et al., 2013a, b; Graham et al., 2013; Shirazi et al., 2013; Harasta et al., 2015; Suchankova et al., 2015; Sørensen et al., 2015; Sirohi et al., 2016; Vallöf et al., 2016, 2019b, 2020; Fortin and Roitman, 2017; Tuesta et al., 2017; Bornebusch et al., 2019; Zhang et al., 2020). Here, we described, for the first time, that liraglutide and semaglutide had similar transient inhibitory effects on EtOH intake, but different effects on EtOH preference. In humans, liraglutide and semaglutide have an estimated half-life of 13 and 165 h, respectively (Agersø et al., 2002; Kapitza et al., 2015). Because of its longer half-life and long-acting properties, semaglutide is injected once weekly, as opposed to liraglutide which is injected once daily (Hall et al., 2018). In animals, liraglutide and semaglutide have an estimated half-life of 23 and 64 h, respectively, after subcutaneous administration (Lau et al., 2015). Although previous studies have investigated acute administration of liraglutide, they only reported its effects over a 24-h period, leaving room for speculation about possible time course and persistence of its effect on EtOH intake (Shirazi et al., 2013; Sirohi et al., 2016; Vallöf et al., 2016). The lack of long-lasting effects of GLP-1 analogs on EtOH intake after acute injection could be due to a faster metabolism of GLP-1 analogs in EtOH drinking animals and/or the need for repeated injections. In support of this hypothesis, long-lasting effects of liraglutide and dulaglutide on EtOH intake have been demonstrated after their daily administration for several consecutive weeks (Vallöf et al., 2016, 2020). However, relatively long-lasting effects of GLP-1 analogs on body weight were observed in the present study. Altogether, these findings suggest that the suppressing effects of GLP-1 analogs on EtOH intake and body weight may be mediated through different mechanisms – a question beyond the scope of the present study.

Malaise and nausea are common side effects of GLP-1 analogs (Buse et al., 2009; Hall et al., 2018), and could be, at least partially, responsible for their effects on EtOH intake, food intake, and body weight. Indeed, concentration higher than 50 μg/kg (i.p.) of liraglutide induces conditioned taste aversion (CTA) (Kanoski et al., 2012). However, lower dose of liraglutide (10 μg/kg, i.p.) has been found to reduce chow consumption without inducing CTA, suggesting that nausea does not solely account for the effects of higher concentration of liraglutide on food intake (Kanoski et al., 2012). In this study, we found that liraglutide significantly decreased EtOH intake without affecting EtOH preference on the day of injection. However, liraglutide significantly decreased EtOH preference at 2-day post-injection, while concomitantly increasing water intake at this time-point. These data tentatively suggest that liraglutide’s effects may not be selective for EtOH. Indeed, liraglutide has been shown to affect water intake after peritoneal injection (McKay et al., 2011). In contrast to liraglutide, semaglutide significantly decreased EtOH preference and did not affect water intake, suggesting that its effects are more selective for EtOH. Consistent with our findings, previous studies suggest that the effects of GLP-1 analogs in reducing EtOH intake are due to reduced EtOH rewarding properties rather than nausea, which is a common side effect of GLP-1 analogs (Buse et al., 2009; Egecioglu et al., 2013b; Shirazi et al., 2013; Sirohi et al., 2016; Vallöf et al., 2016, 2019a, 2020; Hall et al., 2018). Altogether, our results suggest that semaglutide is more specific for alcohol than liraglutide, and given its more favorable prescription dosing (i.e., once a week), may be better suited for use in patients with AUD. However, clinical trials will be needed to test and compare the safety, efficacy, and patients’ compliance to semaglutide and liraglutide as AUD treatment options.

Previous findings that the inhibitory effect of exendin-4 on EtOH intake was blunted in mice lacking GLP-1Rs in the CNS suggest that central GLP-1Rs play a key role in mediating the effects of GLP-1 analogs on EtOH intake (Sirohi et al., 2016). A recent study demonstrated that microinjection of Ex9-39 into the NTS was able to prevent the suppressing effect of exendin-4 on EtOH-induced increase in locomotor activity (Vallöf et al., 2019a). Here, we showed that co-administration of Ex9-39 with liraglutide or semaglutide did not prevent the reduction in EtOH intake and weight. The lack of inhibitory effects of Ex9-39 on GLP-1 analog actions could be due to differences in the half-lives of the drugs. While the exact metabolism profile of Ex9-39 is unclear, it is expected that its half-life would be similar to that of its parent peptide, exendin-4 (Göke et al., 1993), which is 33 min after intravenous and 2.5 h after subcutaneous administration in humans (Edwards et al., 1999; Kolterman et al., 2005). The putatively shorter half-life of Ex9-39, compared to both liraglutide and semaglutide (see above), could provide an explanation for the lack of effectiveness of Ex9-39 in blocking the effects of GLP-1 analogs in this study. The lack of inhibitory effect of Ex9-39 could also be due to insufficient blockade of central GLP-1Rs, which are critical in mediating reduced EtOH intake (Sirohi et al., 2016). Because Ex9-39 was administered intraperitoneally, it is possible that Ex9-39 had limited access to the brain leading to insufficient inhibition of central GLP-1Rs. A previous study showed that Ex9-39 administration increased EtOH intake (Shirazi et al., 2013). Here, we found that Ex9-39, administered alone, did not affect EtOH intake, which may also suggest a lack of relative effectiveness of the drug, at least in the context of our experiments. The discrepancy in the effects of Ex9-39 in Shirazi et al. (2013) and our study could also be due to differences in experimental designs. In our study, each rat was its own control (within-subject design); therefore, the EtOH/water intake over the 24-h period after injection was directly compared to each subject’s basal intake. In contrast, the between-subject comparisons performed by Shirazi et al. (2013) compared EtOH/water intake from two different groups of animals. The within-subject design of this study allowed us to control the potential confounding factors and account for individual differences in baseline EtOH consumption. Additional studies are needed to further investigate the effects of systemic GLP-1R antagonism and its effectiveness in blocking central GLP-1R-mediated effects on alcohol consummatory behaviors.

Several studies have started to unravel the putative neuronal networks and cellular pathways underlying the beneficial effects of GLP-1 analogs in reducing alcohol intake. Initially, GLP-1 analogs were thought to cross the blood brain barrier (BBB), but increasing evidence suggest that this may not be the case, and instead, they may reach the CNS via the circumventricular organs (CVOs), characterized by loose and permeable BBB (Kastin and Akerstrom, 2003; Hunter and Hölscher, 2012; Secher et al., 2014; Ast et al., 2020; Gabery et al., 2020). The effects of GLP-1 analogs on alcohol-related behaviors have been attributed to the activation of central, not peripheral, GLP-1Rs (Sirohi et al., 2016). GLP-1Rs are expressed in brain areas involved in the development and maintenance of AUD, such as NAc, VTA, amygdala, and paraventricular nucleus of the hypothalamus (PVN) (Cork et al., 2015; Graham et al., 2020). Activation of GLP-1Rs has been shown to modulate the increased activity of the mesolimbic dopamine system induced by alcohol (Egecioglu et al., 2013b; Vallöf et al., 2016, 2019a). Peripheral administration of liraglutide prevents alcohol-induced increase in dopamine release in the NAc in rats (Vallöf et al., 2016), while microinjection of exendin-4 into the NAc shell was shown to reduce EtOH intake and block EtOH-induced increase in locomotor activity (Vallöf et al., 2019b). These findings suggest that the effects of GLP-1 analogs on alcohol consumption may result from decreased alcohol-induced dopamine release in the NAc, which in turn, attenuates the rewarding properties of alcohol.

Recent studies have highlighted the importance of NTS GLP-1Rs in mediating the effects of GLP-1 analogs. Selective knockdown of NTS GLP-1Rs attenuates liraglutide-induced decreases in food intake and body weight (Fortin et al., 2020). Moreover, local activation of NTS GLP-1Rs decreases EtOH intake and prevents EtOH-induced accumbal dopamine release (Vallöf et al., 2019a). Located in the brainstem, the NTS is a key regulator of the gut-brain axis, the immune system, and the integration of autonomic functions (Andresen and Kunze, 1994; Marty et al., 2008; Zoccal et al., 2014). The NTS is the primary source of GLP-1-producing neurons, also known as preproglucagon (PPG) neurons, in the CNS, which are believed to play a critical role in addictive behaviors, as they project to and receive inputs from several regions involved in reward processing and consummatory behaviors (Larsen et al., 1997; Merchenthaler et al., 1999; Llewellyn-Smith et al., 2011; Holt et al., 2019). Selective activation of NTS PPG neurons, using chemogenetic techniques, has been shown to mimic the suppressing effects of GLP-1 on food consumption (Gaykema et al., 2017; Holt et al., 2019). Additional optogenetic studies found that the NTS-to-PVN GLP-1 neural pathway is critical for anorectic properties of PPG neurons (Liu et al., 2017). Altogether, these findings suggest that the effects of GLP-1 analogs on alcohol intake may be mediated by NTS GLP-1R-expressing neurons, while their effects on food intake may be mediated by NTS PPG neurons, which could also explain the transient vs. long-lasting effects of liraglutide and semaglutide on EtOH intake vs. body weight, respectively.

Due to the difficulties in developing orally bioavailable non-peptide agonists at GLP-1Rs, and class B GPCRs of the secretin family in general, numerous studies have focused their interest on identifying novel class A rhodopsin-like GPCRs with similar pancreatic β-cells expression and G-protein coupling to GLP-1R. From this effort, GPR119 emerged as a promising therapeutic target for the development of orally active, non-peptide, small-molecule agonists to treat type 2 diabetes and obesity (Koole et al., 2010; Yoshida et al., 2010; Semple et al., 2011, 2012; Melancon et al., 2012; Hothersall et al., 2015; Spasov et al., 2017; Fang et al., 2020a). Administration of GPR119 agonists has been shown to increase plasma GLP-1 and insulin, resulting in improved glucose homeostasis in rodents and humans (Chu et al., 2008; Hansen et al., 2011; Katz et al., 2011; Mandøe et al., 2015; Han et al., 2018; Matsumoto et al., 2018). Here, we tested two different synthetic GPR119 agonists. In contrast to GLP-1 analogs, GPR119 agonists failed to reduce EtOH intake and body weight. Several explanations could account for the lack of effectiveness of GPR199 agonists in the present study. Since we did not measure blood GLP-1 levels, it is possible that the injections of GPR119 agonists did not lead to physiologically relevant increases in blood GLP-1. Because of the relatively short half-lives of AR231453 and APD668, estimated at around 1 h after oral and intravenous administration, respectively, it is also possible that GPR119 agonists may not be able to produce sustained increases in endogenous GLP-1 levels necessary to mimic the effects of GLP-1 analogs (Chu et al., 2008; Semple et al., 2008, 2011). In support of this hypothesis, studies have demonstrated that co-administration of GPR119 agonists with a DPP-4 inhibitor, to prevent rapid degradation of GLP-1, allows for a greater and sustained increase in blood GLP-1 and enhanced effects on glucose homeostasis (Chu et al., 2008; Bahirat et al., 2017; Park et al., 2017). Although we showed that inhibition of DPP-4 with sitagliptin did not affect EtOH intake, co-injection of DPP-4 with GPR119 agonists could be more effective in increasing endogenous GLP-1 levels (Chu et al., 2008) and potentially influencing EtOH drinking. Future studies will be needed to investigate the effects of newly developed compounds with both potent DPP-4 inhibition and GPR119 agonistic activity (Fang et al., 2020b; Li et al., 2020). It is also possible that exposure to alcohol via the IA2BC paradigm may damage GLP-1 producing intestinal L-cells. Alcohol administration was shown to induce intestinal hyperpermeability and endotoxemia (Keshavarzian et al., 2009), which could result in deficient release of endogenous GLP-1 and subsequently blunt the potential effects of endogenous GLP-1 stimulators on alcohol-related outcomes.

In summary, findings of the present study suggest that, among different classes of drugs with modulatory effects on the GLP-1 system, GLP-1 analogs hold the strongest promise to be effective in reducing alcohol consumption, and semaglutide may overall be a better drug candidate than liraglutide in this regard. Preclinical studies are still needed to further characterize the cellular mechanisms and neuronal circuitry which mediate the suppressant effects of GLP-1 analogs on alcohol intake. It is also important to note that chronic exposure to alcohol and alcohol dependence lead to dysregulation of several neural circuits, such as the mesolimbic dopamine and the corticotropin releasing factor systems (Diana et al., 1993; Weiss et al., 1996; Volkow et al., 2007; Roberto et al., 2010; Marty and Spigelman, 2012; Liang et al., 2014a, b; Herman et al., 2016; Marty et al., 2020), which could alter the beneficial effects of GLP-1 analogs observed in non-dependent subjects – an important question that should be explored in future studies. Overall, the present study adds to the growing literature suggesting that the GLP-1 receptor could be a novel and promising pharmacotherapeutic target for AUD. If the results are confirmed in proof-of-concept human studies, repurposing of the readily available FDA-approved medications acting on the GLP-1 system would allow for a quick and efficient medication development process to add new options to the armamentarium of pharmacotherapies for AUD.

## Data Availability Statement

The original contributions presented in the study are included in the article/Supplementary Material, further inquiries can be directed to the corresponding author/s.

## Ethics Statement

The animal study was reviewed and approved by the University of California, Los Angeles, Animal Research Committee.

## Author Contributions

VM, MF, LL, and IS designed the experiments. VM, JM, and YM conducted the experiments and analyzed the data. VM prepared and wrote the manuscript. MF, LL, and IS prepared and edited the manuscript. LL and IS supervised the project. All authors contributed to the article and approved the submitted version.

## Conflict of Interest

The authors declare that the research was conducted in the absence of any commercial or financial relationships that could be construed as a potential conflict of interest.
