# Supplementary material for: Long-Acting Glucagon-Like Peptide-1 Receptor Agonists Suppress Voluntary Alcohol Intake in Male Wistar Rats
Source: Front Neurosci. 2020 Dec 23;14:599646. doi: 10.3389/fnins.2020.599646 (PMC7785877; doi:10.3389/fnins.2020.599646)
Supplement: Supplementary file 1 [file Table_1.DOCX]

**Supplementary Material**

**Table S1**: Two-way RM ANOVA analyses on EtOH intake.

|  | Treatment^1^ | Time-point^2^ | Treatment × Time-point |
| --- | --- | --- | --- |
| AR231453 | F(1,11)=0.19, p=0.67 | F(2,22)=0.55, p=0.58 | F(2,22)=1.89, p=0.17 |
| APD668 (DMSO) | F(1,11)=1.15, p=0.31 | F(2,22)=2.98, p=0.07 | F(2,22)=5.75, p=0.009 |
| APD668 (PEG) | F(1,11)=1.96, p=0.19 | F(2,22)=0.67, p=0.52 | F(2,22)=2.79, p=0.08 |
| liraglutide | F(1,11)=16.41, p=0.002 | F(2,22)=52.72, p<0.001 | F(2,22)=47.5, p<0.001 |
| semaglutide | F(1,11)=14.49, p=0.003 | F(2,22)=72.65, p<0.001 | F(2,22)=65.75, p<0.001 |
| lira+Ex9-39 | F(1,11)=30.73, p<0.001 | F(2,22)=63.26, p<0.001 | F(2,22)=49.22, p<0.001 |
| sema+Ex9-39 | F(1,11)=19.72, p<0.001 | F(2,22)=100.9, p<0.001 | F(2,22)=62.89, p<0.001 |
| Sitagliptin | F(1,11)=7.45, p=0.019 | F(2,22)=7.95, p=0.0025 | F(2,22)=0.95, p=0.4 |
| Ex9-39 | F(1,11)=12.93, p=0.004 | F(2,22)=2.65, p=0.09 | F(2,22)=3.8, p=0.038 |

^1^ Treatment: drug vs. vehicle

^2^ Time-point: baseline, injection day, +2 days

**Table S2**: Two-way RM ANOVA analyses on EtOH preference.

|  | Treatment^1^ | Time-point^2^ | Treatment × Time-point |
| --- | --- | --- | --- |
| AR231453 | F(1,11)=0.56, p=0.47 | F(2,22)=1.5, p=0.24 | F(2,22)=0.14, p=0.87 |
| APD668 (DMSO) | F(1,11)=5.05, p=0.046 | F(2,22)=0.04, p=0.86 | F(2,22)=2.49, p=0.11 |
| APD668 (PEG) | F(1,11)=12.4, p=0.005 | F(2,22)=3.6, p=0.044 | F(2,22)=1.77, p=0.19 |
| liraglutide | F(1,11)=0.83, p=0.38 | F(2,22)=2.27, p=0.13 | F(2,22)=4.29, p=0.03 |
| semaglutide | F(1,11)=1.6, p=0.23 | F(2,22)=20.78, p<0.001 | F(2,22)=14.01, p<0.001 |
| lira+Ex9-39 | F(1,11)=0.1, p=0.75 | F(2,22)=1.07, p=0.36 | F(2,22)=3.75, p=0.04 |
| sema+Ex9-39 | F(1,11)=1.01, p=0.34 | F(2,22)=5.78, p=0.009 | F(2,22)=3.8, p=0.037 |
| Sitagliptin | F(1,11)=11.48, p=0.006 | F(2,22)=0.82, p=0.45 | F(2,22)=0.55, p=0.58 |
| Ex9-39 | F(1,11)=0.0008, p=0.93 | F(2,22)=1.2, p=0.32 | F(2,22)=0.19, p=0.82 |

^1^ Treatment: drug vs. vehicle

^2^ Time-point: baseline, injection day, +2 days

**Table S3**: Two-way RM ANOVA analyses on water intake.

|  | Treatment^1^ | Time-point^2^ | Treatment × Time-point |
| --- | --- | --- | --- |
| AR231453 | F(1,11)=1.65, p=0.22 | F(2,22)=3.01, p=0.07 | F(2,22)=0.18, p=0.83 |
| APD668 (DMSO) | F(1,11)=17.1, p=0.002 | F(2,22)=0.16, p=0.85 | F(2,22)=2.85, p=0.08 |
| APD668 (PEG) | F(1,11)=43.43, p<0.001 | F(2,22)=4.33 p=0.026 | F(2,22)=2.44, p=0.11 |
| liraglutide | F(1,11)=10.78, p=0.007 | F(2,22)=14.06, p<0.001 | F(2,22)=17.78, p<0.001 |
| semaglutide | F(1,11)=6.8, p=0.02 | F(2,22)=0.39, p=0.68 | F(2,22)=2.03, p=0.16 |
| lira+Ex9-39 | F(1,11)=13.36, p=0.004 | F(2,22)=16.25, p<0.001 | F(2,22)=12.04, p<0.001 |
| sema+Ex9-39 | F(1,11)=19.02, p=0.001 | F(2,22)=0.0005, p>0.99 | F(2,22)=1.12, p=0.35 |
| Sitagliptin | F(1,11)=44.53, p<0.001 | F(2,22)=1.15, p=0.33 | F(2,22)=1.32, p=0.29 |
| Ex9-39 | F(1,11)=1.15, p=0.31 | F(2,22)=2.75, p=0.09 | F(2,22)=0.01, p=0.99 |

^1^ Treatment: drug vs. vehicle

^2^ Time-point: baseline, injection day, +2 days
